# Endonuclease from Gram-Negative Bacteria *Serratia marcescens* Is as Effective as Pulmozyme in the Hydrolysis of DNA in Sputum

**DOI:** 10.3389/fphar.2018.00114

**Published:** 2018-02-16

**Authors:** Gulnaz Vafina, Elmira Zainutdinova, Emil Bulatov, Maria N. Filimonova

**Affiliations:** Institute of Fundamental Medicine and Biology, Kazan Federal University, Kazan, Russia

**Keywords:** *Serratia marcescens* nuclease, Sma nuc, Pulmozyme, hydrolysis of DNA, sputum

## Abstract

One of the approaches to effective airway cleansing is the degradation of DNA into smaller fragments. For this purpose Pulmozyme^®^ is used with high efficacy because it contains recombinant DNase I as its active component. The aim of the study was to comparatively analyze DNase activity of Pulmozyme^®^ and the nuclease from gram-negative bacteria *Serratia marcescens*, because at optimal conditions the catalytic efficiency of the nuclease is much higher than the efficiency of DNase I. Highly polymerized DNA and purulent-mucous sputum were used as substrates. The examination showed that both *S. marcescens* nuclease and Pulmozyme^®^ hydrolyzed DNA in sputum. Also *S. marcescens* nuclease was found capable of hydrolyzing DNA in conditions that are standard for Pulmozyme^®^ and suitable for its therapeutic application. For manifesting the similar hydrolytic activity the nuclease amount in the assay mixture containing highly polymerized DNA or the sonicated sputum and NaCl together with calcium- or magnesium- cations can be about 10- time lower than that of the recombinant DNase I. In the presence of magnesium cations the DNase activity of both *S. marcescens* nuclease and Pulmozyme^®^ was higher than in the presence of calcium cations.

## Introduction

*Serratia marcescens* nuclease (EC 3.1.30.2) originates from Gram negative bacteria *S. marcescens* and heads a family of homological non-specific nucleases that are widely spread in the world (Chen et al., 2009). *S. marcescens* nuclease is the most studied nuclease in this family. Its cultivation features, structure, mechanisms of action, as well as some physical, chemical, and biochemical properties are well-studied (Nestle and Roberts, 1969; Leshchinskaya et al., 1974; Biedermann et al., 1989; Pedersen et al., 1993; Miller et al., 1994; Filimonova et al., 1996, 1997, 2001, 2003, 2014; Friedhoff et al., 1996a,b; Benedik and Strych, 1998; Shlyapnikov et al., 2000; Berkmen and Benedik, 2002; Miller and Krause, 2008; Romanova and Filimonova, 2012; Trifonova et al., 2015; Romanova et al., 2016) and represented in such famous data banks as SCOP, RCSB, Brenda. The nuclease has broad utility due to its potent digestive activity toward both DNA and RNA. At optimal conditions the catalytic efficiency of *S. marcescens* nuclease is more than 30-times greater than the efficiency of DNase I (Benedik and Strych, 1998). The hydrolytic activity of the nuclease is enhanced in the presence of magnesium cations (Nestle and Roberts, 1969; Leshchinskaya et al., 1974; Filimonova et al., 1997; Romanova et al., 2016). However, for manifesting the high hydrolytic activity in *S. marcescens* nuclease the ratio of magnesium cations to phosphate groups in DNA is more important than the concentration of these cations by their self (Filimonova et al., 1997).

Pulmozyme^®^ (“F. Hoffmann-La Roche AG,” Switzerland) is a medicinal preparation containing Dornase alpha as its active component. Dornase alpha is a recombinant human deoxyribonuclease I, represented by a glycoprotein with a molecular weight of about 37 kDa. Due to its ability to degrade DNA, during studies Dornase alpha caused hydrolysis of extracellular DNA in purulent sputum of patients, significantly reduced its visco-elastic properties and improved mucociliary clearance (Voronkova et al., 2006).

Sputum is a pathological secretion formed by diseases of the trachea, bronchial tree, and lung tissue. Depending on the type and severity of the pathological process, the quantity and qualitative composition of the sputum varies. So in purulent and mucopurulent sputum a large amount of neutrophils and their residues are observed. Neutrophils phagocytose both bacteria and degradation products in tissue and destroy them with their lysosomal enzymes (proteases, peptidases, oxidases, deoxyribonucleases, and lipases) (Gullberg et al., 1997). The death of neutrophils in response to infection is accompanied by the release of mucoid glycoproteins and deoxyribonucleic acid (DNA). The accumulation of neutrophils and their death leads to accumulation of DNA and mucoid glycoproteins, which increases sputum viscosity, significantly impairs drainage of the broncho-pulmonary system that facilitates the development of infection. The latter leads to a further release of neutrophils and the formation of even more DNA and mucoid glycoproteins.

One of the approaches to effective airway cleansing is the degradation of DNA into smaller fragments. The high efficacy of Pulmozyme^®^ have been established for treatment of pulmonary diseases such as chronic bronchitis, Kartagener’s syndrome, chronic obstructive pulmonary disease, lobar atelectasis, bronchiectasis, and cystic fibrosis.

The aim of this study is to comparatively analyze DNase activity of Pulmozyme^®^ and the extracellular endonuclease from gram-negative bacteria *S. marcescens* toward the substrate, purulent-mucous sputum.

## Materials and Methods

All reagents were purchased from commercial sources, unless stated otherwise – magnesium sulfate, calcium chloride, and DNA from herring testes (type XIV) were purchased from Sigma-Aldrich. All the described reagents were used without further purification. Bacterial strain W1050 of *S. marcescens* was kindly provided by Prof. Michael Benedik (University of Houston, United States). A commercial preparation Pulmozyme^®^ (F. Hoffman-La Roche) contained 1 mg Dornase alfa, 8.77 mg NaCl, and 0.152 mg CaCl_2_⋅2H_2_O per 1 ml.

### Purification of *S. marcescens* Nuclease

It was carried out as was described earlier (Vafina et al., 2016). The procedure was based on routine preparation of the culture medium, and fractional salt precipitation of the crude nuclease, the dialysis, and cation exchange chromatography with NGC Discover chromatography system using UNO S12 column (Bio-Rad, United States). The DNA-degrading enzymatic activity in the collected fractions was tested as previously described (Leshchinskaya et al., 1974). The purity and the apparent molecular mass of proteins from the peak fractions were analyzed by denaturing SDS-PAGE using 12% gel.

### Preparation and Characterization of Sputum

#### Sputum of a Patient

A patient diagnosed with purulent-mucous bronchitis were taken into glycerol and stored at -25°C. All subjects gave written informed consent in accordance with the Declaration of Helsinki. The protocol was approved by Ethic Expert Committee of Kazan Federal University, Republic of Tatarstan, Russian Federation.

Before using the glycerol was removed. For this purpose the mixture of sputum with glycerol was mixed with a twofold volume of 0.9% NaCl and, after mixing, the sputum was collected by 25 min centrifugation at 9,000 rpm with cooling. The supernatant was decanted. A threefold volume of 0.9% NaCl was added to the precipitate and the described above procedure was repeated twice. The resulting precipitate was then mixed with an equal volume of 0.9% NaCl and sonicated twice with cooling in an ice-water bath for five 5-s periods in Sonopuls 2200 (Bandelin) with a 3 mm titanium probe and an energy setting of 100 W until visual uniformity was achieved.

#### Determination of Nucleic Acids and Proteins in the Sonicated Sputum by UV Spectroscopy

The A_280_, A_260_, and A_235_ were measured with the sonicated sputum diluted 100-fold with distilled water. The solvent blank containing distilled water was used to zero the instrument at each wavelength. The nucleic acid amount was found on the basis of A_280_/A_260_ in accordance with Warburg and Christian (1942). Calculations of the protein amount (mg/ml) were done in respect with the equations of protein concentration mg/ml = (A_235_-A_280_)/2.51 (Whitaker and Granum, 1980) and = 1.45 A_280_-0.74 A_260_ (Kalckar, 1947). Visualization of proteins in sonicated sputum was performed with standard Coomassie Brilliant Blue staining protocol for the polyacrylamide gels that was applied for the agarose gel after visualization of DNA in the gels with ethidium bromide staining.

#### Evaluation of DNA Polymericity

Evaluation of DNA polymericity in the sonicated sputum were made with agarose gel electrophoresis with routine conditions (Wegrzyn et al., 1991) and, also, with perchloric acid on the basis of Nestle and Roberts (1969) and Leshchinskaya et al. (1974). The equivalent volume of the chilled 4% perchloric acid was added to the sonicated sputum 100-fold dissolved with distilled water. After 15 min incubation at the ice bath the precipitate of highly polymerized DNA was removed by centrifugation. The absorption of supernatant was monitored at 260 nm. The DNA amount (mg/ml) dissolved with perchloric acid was calculated on the basis of A_260_/23 (Johnson, 1994).

### Levels of the DNase Activity

DNase activity of Pulmozyme^®^ and *S. marcescens* nuclease were determined using 96-well plate assay modified from that described previously (Ball et al., 1990). The wells of microwell dish were filled with 100 μl of the assay mixture that was composed of 10-fold diluted with 0.9% NaCl sonicated sputum (see above) and either 0.9% NaCl, containing 0.002 M MgSO_4_⋅7H_2_O or 0.9% NaCl containing 0.002 M CaCl_2_⋅2H_2_O at the ratio of 5:4. The 1 ml of the assay mixture also contained 10 μg of ethidium bromide. A 50 μl sample of the tested enzyme was respectively applied to the top well of each column and 50 μl was removed and added to the next well. These threefold dilutions were continued for the eight wells in the column. Dilutions of all eight samples were made concurrently by using an eight tips multichannel pipette. 0.9% NaCl solution was used instead of tested enzymes for the control sample (first and fifths columns of each plate). Each sample of the tested enzymes was applied with the aim of reproducibility to the tops of three columns (three repetitions) – 2nd, 3rd, 4th for Pulmozyme^®^ or 6th, 7th, 8th for the nuclease. The plate was covered with the foil and incubated at 37°C. After incubation for 1–90 min the plate was placed on a UV light box and photographed. The loss of fluorescence due to DNA degradation was visualized and each sample was compared with standard solution in the first and fifths columns of each plate.

### For the Quantitative Comparative Analysis

The DNase activity of Pulmozyme^®^ and *S. marcescens* nuclease was assayed by the method modified from that previously described (Leshchinskaya et al., 1974). The assay mixture included 0.36% NaCl or 0.36% NaCl containing 0.8 mM CaCl_2_⋅2H_2_O or 0.36% NaCl containing 0.8 mM MgSO_4_⋅7H_2_O instead 80 mM Tris-HCl buffer, pH 8.5, containing 7 mM MgSO_4_⋅7H_2_O, and highly polymerized DNA (1 mg/ml) or the sonicated sputum (respectively twofold diluted in the assay mixture). The incubation was performed at 37°C for 15 min when the assay mixture contained highly polymerized DNA or 1–10 min when the assay mixture contained the sonicated sputum. The enzymes were used without dilution toward the substrate, sonicated sputum, or were respectively diluted with the water so that about 15–50% of highly polymerized DNA was converted to acid-soluble products. In particular when the assay mixture contained highly polymerized DNA and NaCl, or NaCl with calcium, or NaCl with magnesium the nuclease was 10- or 5- or 20-times diluted, and Pulmozyme^®^ was respectively 5- or 4- or 6 times diluted before addition to the assay mixture. Each experiment was repeated not less than three times when highly polymerized DNA was used or 2–4 times toward the sonicated sputum.

## Results and Discussion

### Purification of *S. marcescens* Nuclease

Using the earlier published protocol for purification of *S. marcescens* nuclease (Vafina et al., 2016) we obtained the purified enzyme exhibiting high level of activity. The results of SDS-PAGE together with enzymatic activity measurements confirmed high purity of the isolated nuclease and validated its identity. Since, we knew that 1 mg of the homogeneous enzyme corresponded to 1,600,000 units of the DNase activity defined in accordance with (Leshchinskaya et al., 1974) we assumed that the chosen preparation contained 0.103 mg of the nuclease protein per ml. This preparation was further used for comparative analysis of hydrolytic activity of the purified nuclease and Pulmozyme^®^ toward the highly polymerized DNA and nucleic acids in the sputum.

### Characterization of the Sputum

**FIGURE 1:**
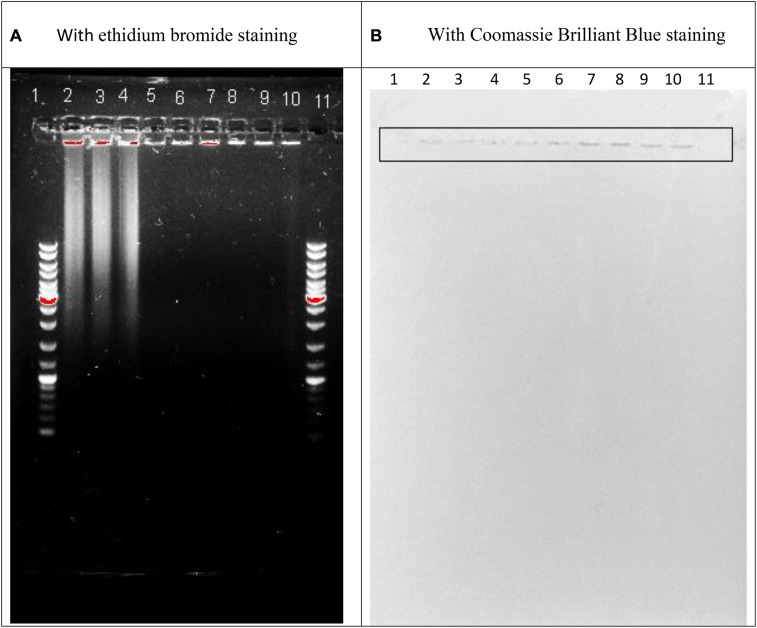
Agarose gel electrophoresis of the sonicated sputum containing 0.36% NaCl (lines 2, 5, 8) or 0.36% NaCl together with 0.8 mM MgSO_4_⋅7H_2_O (lines 3, 6, 9) or 0.36% NaCl together with 0.8 mM CaCl_2_⋅2H_2_O (lines 4, 7, 10) after 60 min incubation in the absence (lines 2–4) or in the presence of *Serratia marcescens* nuclease (0.01 mg in 1 ml of the assay mixture; lines 8–10) or Pulmozyme^®^ (0.1 mg in 1 ml of the assay mixture; lines 5–7) and the diagnostic staining with ethidium bromide **(A)** or Coomassie Brilliant Blue **(B)**. Lines 1 and 11 are GeneRuler DNA Ladder Mix SM0331 for Nucleic Acid Gel Electrophoresis and Blotting (Thermo Scientific TM, containing the fragment of 10,000; 8,000; 6,000; 5,000; 4,000; 3,500; 3,000; 2,500; 2,000; 1,500; 1,200; 1,000; 900; 800; 700; 600; 500; 400; 300; 200; 100 base pairs).

The A_280_, and A_260_, and A_235_ of the sonicated sputum 100-fold dissolved with distilled water were respectively 0.398 ± 0.015, 0.432 ± 0.014; 0.686 ± 0.020. The protein calculation with (Whitaker and Granum, 1980) or with (Kalckar, 1947) showed its amount, respectively 11.46 ± 0.4 or 25.8 ± 1.2 mg/ml. The nucleic acids amount was found on the basis of the proportions (Warburg and Christian, 1942). As it varied between 4 and 5% we summarized that the nucleic acid amount in the sonicated sputum was at the level of 0.44–1.35 mg/ml. Addition of 4% perchloric acid to the sonicated sputum caused decreased absorption of the resulted supernatant that confirmed a presence of the polymeric deoxyribonucleotides. The picture of agarose gel electrophoresis of the sonicated sputum is shown in **Figure 1A**. As seen from **Figure 1A**, the sonicated sputum contained the fragments of 3 kb and over that verified the polymericity of DNA fragments in the sonicated sputum. A part of the examined factions remained at the start. This part could be stained with Coomassie Brilliant Blue (**Figure 1B**), additionally to ethidium bromide staining (**Figure 1A**). The simultaneous staining of the same material with diagnostic dyes for proteins and nucleic acids led us to the conclusion that the material at the start of the gel belonged to nucleoproteins fragments.

### Comparative Analysis of DNase Activity in *S. marcescens* Nuclease and Pulmozyme^®^ toward Highly Polymerized DNA in the Presence of Sodium Chloride as well as Calcium- or Magnesium- Cations

It is known that the DNase activity of Pulmozyme^®^ (Dornase alfa) in the presence of sodium chloride and calcium chloride dihydrate corresponds to 1000 units/mg. It is also known that the activity of *S. marcescens* nuclease depends on the presence of magnesium cations in the medium, which serve as its activators. In this connection, at first, we compared how DNase activity of *S. marcescens* nuclease or Pulmozyme^®^ changed in dependence on the cationic composition of the surrounding medium. The activity was determined by the routine method (Leshchinskaya et al., 1974) with our own modifications. 1 ml of the assay mixture contained as a substrate 1 mg of highly polymerized DNA and NaCl or NaCl with calcium or NaCl with magnesium instead of the buffer solution.

The DNase activity of Pulmozyme^®^ or the nuclease in the presence of sodium chloride with calcium was taken as 100%, as these conditions approximated the conditions of Pulmozyme^®^ action during the therapy.

**FIGURE 2:**
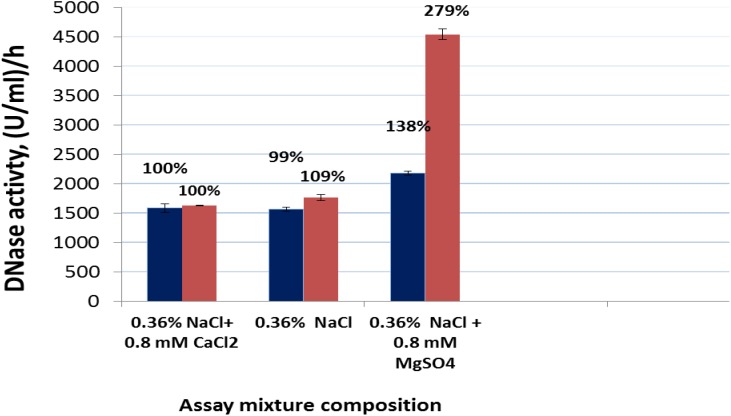
DNase activity of Pulmozyme^®^ (black bars) or *S. marcescens* nuclease (red bars) in the presence or in the absence of calcium chloride or magnesium sulfate in the assay mixture. The assay mixture included highly polymerized DNA (1 mg/ml) and 0.36% NaCl or 0.36% NaCl containing 0.8 mM CaCl_2_⋅2H_2_O or 0.36% NaCl containing 0.8 mM MgSO_4_⋅7H_2_O as well as Pulmozyme^®^ (0.02 or 0.025 or 0.017 mg/ml) or the nuclease (respectively 0.001 or 0.002 or 0.0005 mg/ml).

As seen from **Figure 2** at the tested conditions both enzymes expressed the DNAase activity. The nuclease’s activity in the presence of calcium differed from the Pulmozyme^®^’s activity insignificantly, although the amount of *S. marcescens* nuclease protein in the assay mixture was about 15-fold less than Pulmozyme^®^. At the optimal for *S. marcescens* nuclease conditions, i.e., in the presence of 0.08 M Tris-HCl buffer, pH 8.5, and 8 mM magnesium sulfate, the DNase activity of *S. marcescens* nuclease was about 100-fold higher than that for the tested conditions, i.e., in the presence of sodium chloride with calcium. The lack of calcium chloride in the assay mixture had almost no effect on the DNase activity in *S. marcescens* nuclease or Pulmozyme^®^. Replacement of calcium- by magnesium- cations of the same concentration three-times increased the DNase activity in *S. marcescens* nuclease or by approximately 30% in Pulmozyme^®^.

### Comparative Analysis of DNase Activity in *S. marcescens* Nuclease and Pulmozyme^®^ in Sputum

Since the tested in Section “Comparative Analysis of DNase Activity in *S. marcescens* Nuclease and Pulmozyme^®^ toward Highly Polymerized DNA in the Presence of Sodium Chloride as Well as Calcium- or Magnesium- Cations” conditions were suitable for both Pulmozyme^®^ and *S. marcescens* nuclease to express the hydrolytic activity toward highly polymerized DNA, we have chosen the same conditions for examining the DNase activity of these enzymes in sputum. The assay mixtures contained the sonicated sputum instead highly polymerized DNA and NaCl together with calcium- or with magnesium- cations.

#### Determination of the Level of DNase Activities

**FIGURE 3:**
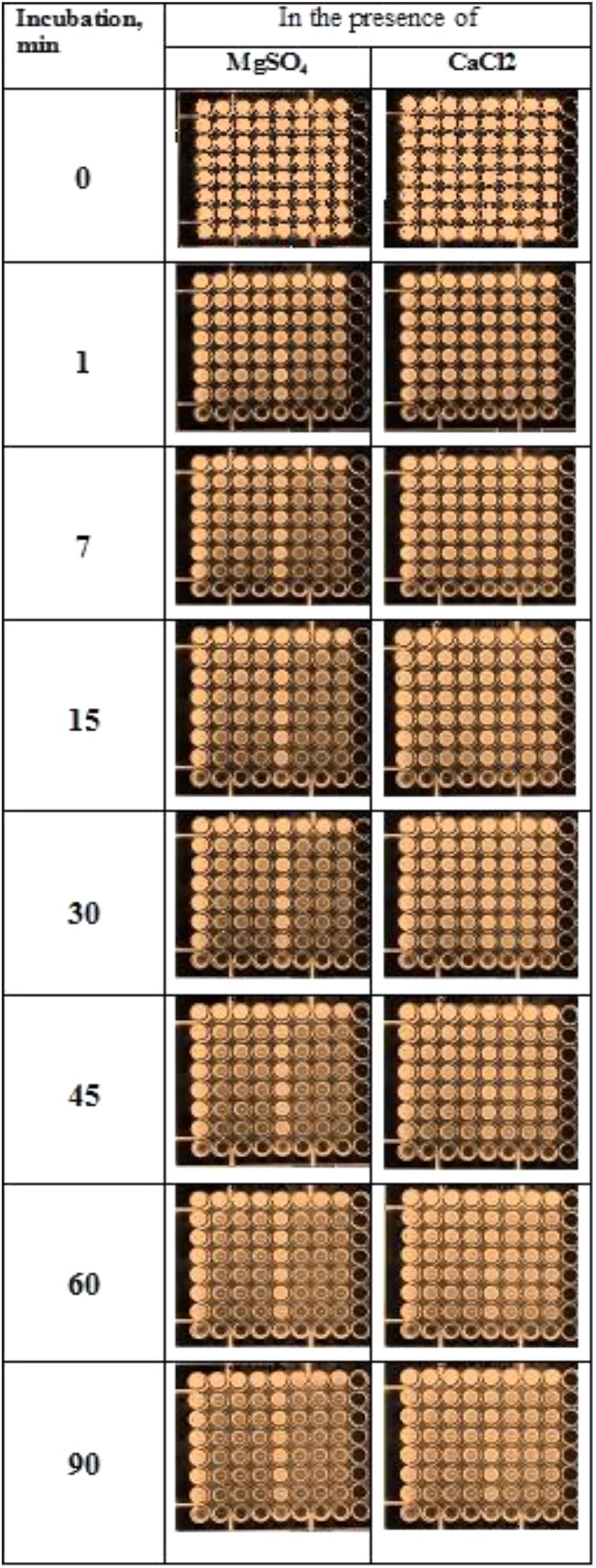
Determination of the levels of the DNase activity of Pulmozyme^®^ (columns 2–4, three repetitions) or *S. marcescens* nuclease (columns 6–8, three repetitions) in the presence of magnesium cations (**left** plates) or calcium cations (**right** plates). The method is based on quenching of luminescence of ethidium bromide incorporated into DNA of sputum in the wells because of DNA degradation with Pulmozyme^®^ or *S. marcescens* nuclease. The amount of Pulmozyme^®^ or *S. marcescens* nuclease in the wells of the first row was respectively 0.33- or 0.034-mg/ml.

Determination of the level of DNase activities in Pulmozyme^®^ and *S. marcescens* nuclease were based on quenching luminescence of ethidium bromide incorporated in DNA. Due to sensitivity of the assay used, the sonicated sputum was 10-fold diluted with 0.9% NaCl before preparation of the assay mixture. The results of determining the level of DNase activities in Pulmozyme^®^ and *S. marcescens* nuclease are shown at **Figure 3**. As seen from the **Figure 3** both Pulmozyme^®^ and *S. marcescens* nuclease degraded DNA in the sputum that was visualized by loss of fluorescence in the wells in the presence of the enzymes (columns 2–4 and 6–8) relative the standard solution, in the absence of enzymes (columns 1 and 5).

In the presence of magnesium cations the hydrolysis of DNA by each enzyme was faster than in the presence of calcium cations (left plate and right plate, correspondingly). In particular, after 45 min incubation in the presence of magnesium a loss of fluorescence was observed in the 7-th row of columns for both Pulmozyme^®^ and the nuclease (left plate, columns 2–4 and 6–8, respectively). The increasing incubation time up to 1.5 h did not affect the pattern of DNA degradation in the presence of magnesium (left plate, the 8-th row). At the initial stage in the presence of magnesium, 1–7 min, the hydrolysis with *S. marcescens* nuclease looked more intensive than with Pulmozyme^®^ (columns 6–8, and 2–4 respectively). On the contrary, in the presence of calcium (right plate), the degradation of DNA by both enzymes was less explicit than in the presence of magnesium (left plate). Additionally the hydrolytic activity of *S. marcescens* nuclease manifested itself weaker than of Pulmozyme^®^ (**Figure 3**, right plate).

Surprisingly, in the control wells at the first row (1st and 5th column in each plate) the loss in fluorescence was also observed. It was already developed after 1 min incubation and did not change for further observation period, 90 min, as it was at the wells containing the compared enzymes. Since it did, we related the quenching fluorescence in the control wells with effect of DNases in sputum, that were in fact the DNases of destroyed neutrophils. The effect of those DNases was apparently small and short-lived, because it observed only in the first row wells of the control columns (1st and 5th column in each plate). Also we suspect that the amount of the sputum DNases or their stability is insufficient for the intensive degradation of sputum DNA.

#### Quantification of the DNase Activities in Pulmozyme^®^ and *S. marcescens* Nuclease toward Sputum

**FIGURE 4:**
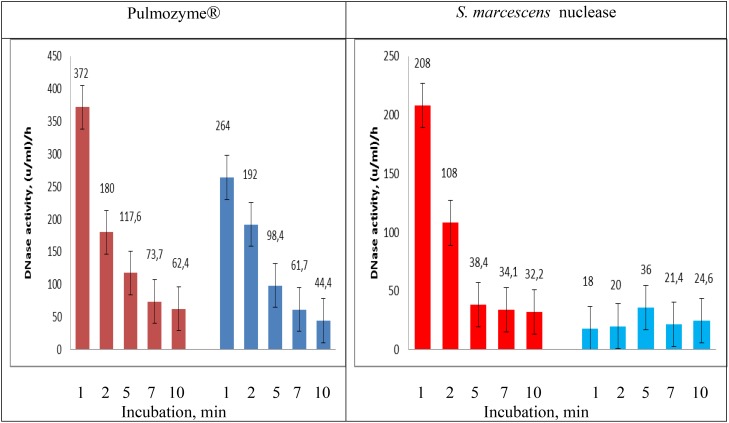
Changing DNAase activity in Pulmozyme^®^ and *S. marcescens* nuclease on sputum in the presence of magnesium – (red bars) or calcium (blue bars) cations. The assay mixtures contained sonicated sputum and 0.36% NaCl together with 0.8 mM CaCl_2_⋅2H_2_O or 0.36% NaCl together with 0.8 mM MgSO_4_⋅7H_2_O as well as Pulmozyme^®^ (0.1 mg/ml) or the nuclease (0.01 mg/ml).

For the quantitative evaluation of the DNase activities in Pulmozyme^®^ and *S. marcescens* nuclease on sputum we used the assay employed during hydrolysis of highly polymerized DNA (see section “Comparative Analysis of DNase Activity in *S. marcescens* Nuclease and Pulmozyme^®^ toward Highly Polymerized DNA in the Presence of Sodium Chloride as Well as Calcium- or Magnesium- Cations”). In order to examine the dynamics in the DNase activities aliquots were taken from the assay mixtures after 1, 2, 5, 7, and 10 min incubations with each of the compared enzymes. The accumulation of the degradation products was determined as described (Leshchinskaya et al., 1974). The results are shown in **Figure 4**. The data in **Figure 4** in general conformed the results that were obtained in both determining the levels of DNase activities on sputum (see section “Determination of the Level of DNase Activities”) and the activities toward highly polymerized DNA (see section “Comparative Analysis of DNase Activity in *S. marcescens* Nuclease and Pulmozyme^®^ toward Highly Polymerized DNA in the Presence of Sodium Chloride as Well as Calcium- or Magnesium- Cations”). Both Pulmozyme^®^ and *S. marcescens* nuclease manifested DNase activity on sputum. The activity of the enzymes in the presence of Mg^2+^ was higher than in the presence of Ca^2+^. However, comparing the results in **Figures 2**, **4** allows to conclude that at the same conditions the hydrolysis of sputum DNA by both enzymes was slower than highly polymerized DNA. This tendency was observed in the presence of both magnesium and calcium cations. Another common tendency observed was the diminishing hydrolytic activity of both enzymes as they incubated. As can be seen from **Figure 4** the activity in both Pulmozyme^®^ and *S. marcescens* nuclease almost fivefold decreased for the 10 min incubation in the presence of magnesium cations. The activity of Pulmozyme^®^ in the presence of calcium- changed likewise it in the presence of magnesium cations. On the contrary the activity of *S. marcescens* nuclease in the presence of calcium- showed a more complicated dependence relative magnesium cations and remained for 10 min at approximately the same level. Since the aim of the undertaken study consisted in comparative analysis of Pulmozyme^®^ and the nuclease from *S. marcescens* by their ability to degrade DNA in sputum we did not examine the cause of diminishing DNase activity in these enzymes during the sputum hydrolysis. The diminishing hydrolytic activity could be connected with either the decreasing substrate concentration or possible digestion of these enzymes by sputum proteases. The idea of inhibition of these enzymes by the hydrolytic products probably may be discarded because previously we had found that any mononucleotides produced by *S. marcescens* nuclease during hydrolysis of DNA or RNA did not influence its activity toward DNA (Romanova and Filimonova, 2012). As the tendency of diminishing DNase activity is common for both Pulmozyme^®^ and the nuclease we conclude that this trend is not connected with the inhibition.

#### Visualization of Degradation DNA in Sputum with *S. marcescens* Nuclease or Pulmozyme^®^

It was carried out using agarose gel electrophoresis. The assay mixtures were composed as described (see section “For the Quantitative Comparative Analysis”) and contained the sonicated sputum and 0.36% NaCl or 0.36% NaCl together with 0.8 mM MgSO_4_⋅7H_2_O or 0.36% NaCl together with 0.8 mM CaCl_2_⋅2H_2_O. After addition at the ratio 1:9 of the undiluted enzymes, *S. marcescens* nuclease or Pulmozyme^®^, or NaCl as a control, it was incubated for 60 min at 37°C. The aliquots were taken from the assay mixtures and used as the samples for the electrophoresis. As shown in **Figure 1A** all examined samples contained DNA that was located at the start position (lines 2–10). The samples incubated without the enzymes additionally contained the DNA material that was located at the level of 3 kb and higher (lines 2–4). And vice versa the samples resulted from incubation with both *S. marcescens* nuclease (lines 8–10) and Pulmozyme^®^ (lines 5–7) were free of such DNA material that was identified by the lack of fluorescence at the respective zone. These data showed that although the DNase activity in both enzymes declined for 10 min incubation in the mixture containing sputum (see section “Quantification of the DNase Activities in Pulmozyme^®^ and *S. marcescens* Nuclease toward Sputum”), it was enough to complete degradation of DNA in sputum for 60 min incubation with both Pulmozyme^®^ and *S. marcescens* nuclease independently on the absence (lines 5 and 8) or the presence of magnesium (lines 6 and 9) or calcium (lines 7 and 10) cations.

Further staining of the same gel with Coomassie Brilliant Blue indicated a protein material in all examined samples (**Figure 1B**). The stained with Coomassie Brilliant Blue bands located at the start position of the gel and matched the bands that were initially indicated with ethidium bromide and belonged to DNA material. This finding allowed suggesting the nucleoprotein material in the sputum.

## Conclusion

Comparative analysis of DNase activities of the nuclease from Gram-negative bacteria *S. marcescens* and Pulmozyme^®^ toward sputum specifies the following.

*Serratia marcescens* nuclease is highly effective on sputum DNA. Its efficiency is comparable with Pulmozyme^®^.

The ability of both enzymes to hydrolyze DNA in sputum was confirmed with forming of acid soluble products of the DNA degradation as well as the quenching luminescence of ethidium bromide initially incorporated in the sputum DNA.

*Serratia marcescens* nuclease is capable to hydrolyze DNA in the presence of NaCl containing calcium cations that are conditions suitable for therapeutic action of Pulmozyme^®^. At the same time for manifesting comparable with Pulmozyme^®^ efficacy the nuclease amount can be several times less than that of Dornase alpha, the active component of Pulmozyme^®^.

## Author Contributions

GV: isolation of *Serratia marcescens* nuclease and analysis of degradation of DNA in sputum. EZ: preparation and characterization of the sonicated sputum. EB: an author of the main idea of testing the nuclease from *Serratia marcescens* for hydrolysis of DNA in sputum. MF: supervision and analysis of all examination. Altogether have given preparation of the manuscript for the publication.

## Conflict of Interest Statement

The authors declare that the research was conducted in the absence of any commercial or financial relationships that could be construed as a potential conflict of interest.
